# Volatile constituents of three *Thymus sipyleus* Boiss. subspecies from different sites in Turkey

**DOI:** 10.3906/kim-2103-6

**Published:** 2021-09-20

**Authors:** Hale Gamze AĞALAR, Mine KÜRKÇÜOGLU, Kemal Hüsnü Can BAŞER, Kenan TURGUT

**Affiliations:** 1 Department of Pharmacognosy, Faculty of Pharmacy, Anadolu University, Eskişehir, Turkey; 2 Department of Pharmacognosy, Faculty of Pharmacy, Near East University, Lefkoşa, North Cyprus; 3 Department of Field Crops, Faculty of Agriculture, Akdeniz University, Antalya, Turkey

**Keywords:** *Thymus sipyleus* subsp. *sipyleus* var. *sipyleus*, *T. sipyleus* subsp. *sipyleus* var. *davisianus*, *T. sipyleus* subsp. *rosulans*, microdistillation, GC and GC-MS analysis, chemical polymorphism, terpenes

## Abstract

This study was designed to reveal the chemical diversity of some *Thymus sipyleus* subspecies growing wild in Turkey and to compare the volatile compound profiles by using micro(hydro)distillation technique. For this purpose, volatile compounds isolated by microdistillation from nine samples (three plant samples collected from different regions in Antalya) of *Thymus sipyleus* Boiss. subsp. *sipyleus* var. *sipyleus*, *T. sipyleus* Boiss. subsp. *sipyleus* var. *davisianus* Ronniger, and *T. sipyleus* Boiss. subsp. *rosulans* (Borbas) Jalas were analyzed by GC and GC-MS systems. 1,8-Cineole, *p*-cymene, α-terpineol and carvacrol were identified as major compounds in *T. sipyleus* subsp. *sipyleus* var. *sipyleus* samples. Geranial, neral, 1,8-cineole and β-caryophyllene, and α-terpineol and geranial were the main compounds in *T. sipyleus* subsp*. sipyleus* var*. davisianus* samples. β-Caryophyllene, intermedeol, 1,8-cineole and α-terpineol, α-pinene were the major compounds in *T. sipyleus* subsp*. rosulans* samples. As known, thymol is the main compound in most *Thymus* species in Turkey, but, according to our study, chemical polymorphism has been found among the *T. sipyleus* subspecies.

## 1. Introduction

The genus *Thymus* is note-worthy among the numerous species and varieties of wild-growing aromatic plants belonging to the family Lamiaceae. Many of these species are typical for the Mediterranean area. The genus *Thymus* is represented by 42 species and 47 taxa, 20 of which are endemic in Turkey [1]. All of them produce essential oils, and only a few are important herbs used in all parts of the world. Most of the terpenoid volatiles detected in *Thymus* oils belong to the monoterpene group. Sesquiterpenes are always present, but with only a few exceptions in minor percentages [2].

Most of these taxa growing in Turkey are aromatic plants which are generally used as herbal tea, condiments and in folk medicine. Carvacrol and thymol are abundant monoterpenes in the essential oils of this genus. However, there are *Thymus* species poor in phenolic compounds and some do not contain phenolic compounds at all. Phenol-rich *Thymus* species are used in diabetes, stomach and intestinal diseases, for cough as herbal tea and also as a condiment; whereas, phenol-poor or phenol-less *Thymus* species are used, due to their pleasant aroma, as herbal tea in Turkey [3].

*Thymus* L. is known in the world as ‘thyme’ and in Anatolia as ‘kekik’ or ‘kaya kekiği’. Volatile oils of thyme are used as antiseptics, antispasmodics and fungicidal [4, 5]. The antiseptic, antioxidative, insecticidal, preservative and anaesthetic properties of thyme are due to their biologically active substances, such as thymol, carvacrol, linalool, geraniol and other volatiles in the essential oil [6]. In addition to the plant applications, thyme oils are also used in flavour and food industries, mainly in the manufacture of perfumes and cosmetics, or for flavouring chocolates, toothpaste, mouthwashes [7].

Due to the high economic value of *Thymus* species, a high number of studies on several aspects of this genus are available as well as the existing monographs on *Thymus* in Pharmacopoeias [8–10].

*Thymus sipyleus* Boiss. is endemic in Turkey, and known with local Turkish names as “kekik, limon kokulu kekik, keklik otu, yayla kekiği, nemamul otu, sater” [11]. According to ethnobotanical records, *T. sipyleus* and its subspecies are used for different purposes. In Adana, infusion of branches and leaves are consumed before meals for the treatment of stomach aches [12]. The aerial parts of *T. sipyleus* subsp. *sipyleus* var. *sipyleus* and *T. sipyleus* subsp. *sipyleus* var. *rosulans* are used as spice and tea (dried and grounded), in the treatment of haemorrhoids, atherosclerosis, and stomach disorders in Osmaneli, Bilecik [13]. The leaves of both subspecies also boiled with lemon as tea are taken against common cold and coughs in Sivas and Yozgat [14]. In Ulukışla, Niğde, an infusion of the aerial parts of *T. sipyleus* subsp. *sipyleus* var. *sipyleus* is consumed three times a day for colds and stomach aches [15]. *T. sipyleus* subsp. *rosulans* known as “catri” in the Eastern part of Turkey is used for diabetes, colds, abdominal ailments as an infusion and decoction [16].

The present study is focused on determining the variation of volatile compounds from different populations of *Thymus sipyleus* Boiss. subsp. *sipyleus* var. *sipyleus*, *T. sipyleus* Boiss. subsp. *sipyleus* var*. davisianus* Ronniger, and *T. sipyleus* Boiss. subsp. *rosulans* (Borbas) Jalas. To date, the oil composition, biological activities of these subspecies of *Thymus sipyleus* have been reported [3, 11, 17–19]. In the present study, microdistilled aerial parts of *T. sipyleus* subsp. *sipyleus* var. *sipyleus*, *T. sipyleus* subsp. *sipyleus*. var*. davisianus*, and *T. sipyleus* subsp. *rosulans* collected from different regions of Antalya, Turkey were analyzed by GC and GC-MS systems, simultaneously. Each microdistilled sample was characterized with major and minor volatile constituents by using in house and commercial libraries.

## 2. Materials and methods

### 2.1. Plant material

Air dried aerial parts of *T. sipyleus* subsp. *sipyleus* var. *sipyleus* (KT:190, 191, 192), *T. sipyleus* subsp*. sipyleus var. davisianus* (KT:196, 197, 198) and *T. sipyleus* subsp*. rosulans* (KT:199, 200, 201) were collected from three regions in Elmalı, Saklıkent, Gazipaşa (Antalya), respectively (Table 1). Identification of plant samples was done by one of us (KT). All herbarium samples coded as KT were kept at the Department of Field Crops, Faculty of Agriculture, Akdeniz University, Antalya, Turkey.

### 2.2. Isolation of the volatiles

Each sample was obtained by microdistillation of the dried, ground plant material (50 mg) using an Eppendorf MicroDistiller with 10 mL distilled water per sample vial. The sample vial was heated to 108 °C at a rate of 20 °C/min and kept at this temperature for 90 min, then heated to 112 °C at a rate of 20 °C/min and kept at this temperature for 30 min. The sample was subjected to a final postrun for 2 min under the same conditions. The collecting vial, containing a solution of NaCl (2.5 g, Sigma-Aldrich) and water (0.5 mL, ultrapure) plus 350 μL of *n*-hexane [Sigma-Aldrich, ≥99% (GC)] to trap volatile components, was cooled to −5 °C during distillation. After the distillation was completed, the organic layer in the collection vial was separated and analyzed by gas chromatography (GC) and gas chromatography-mass spectrometry (GC-MS) systems, simultaneously.

### 2.3. GC analysis

The GC analysis was carried out using an Agilent 6890N GC system. FID detector temperature was 300 °C. To obtain the same elution order with GC-MS, simultaneous autoinjection was done on a duplicate of the same column applying the same operational conditions. Relative percentage amounts of the separated compounds were calculated from FID chromatograms. The results of the analysis are shown in Table 2.

### 2.4. GC-MS analysis

The GC-MS analysis was carried out with an Agilent 5975 GC-MSD system. Innowax FSC column (60 m × 0.25 mm, 0.25 mm film thickness) was used with helium as carrier gas (0.8 mL/min). GC oven temperature was kept at 60 °C for 10 min and programmed to 220 °C at a rate of 4 °C/min, and kept constant at 220 °C for 10 min and then programmed to 240 °C at a rate of 1 °C/min. Split ratio was adjusted at 40:1. The injector temperature was set at 250 °C. Mass spectra were recorded at 70 eV. Mass range was from m/z 35 to 450.

### 2.5. Identification of components

Identification of volatile compounds was carried out by comparison of their relative retention times with those of authentic samples or by comparison of their relative retention indices (RRI) to series of *n*-alkanes (C_8_ to C_25_). Computer matching against commercial (Wiley GC-MS Library, Adams Library, MassFinder 3 Library) and in-house “Başer Library of Essential Oil Constituents” built up by genuine compounds and components of known oils, as well as MS literature data were used for the identification [20].

## 3. Results and discussion

GC and GC-MS analysis of the samples obtained by microdistillation resulted in a total of one hundred fifteen volatile compounds were identified in *Thymus sipyleus* subspecies by using in house and commercial libraries. The elution of the compounds in the microdistilled oils was done by using an HP-Innowax FSC column. Table 2 shows the list of detected and identified volatile constituents with their RRI and relative percentages in the samples.

Seventy seven total components of three *T. sipyleus* subsp*. rosulans* samples were identified by GC-MS. Forty-eight components of the KT199 sample were detected representing 90% of the oil. β-Caryophyllene (14.2%) and intermedeol (13.3%) were the major compounds of this sample. Twenty one volatiles are higher than 1% and other major compounds are 1,8-cineole (8.7%), caryophyllene oxide (6.2%), spathulenol (7.0%), α-humulene (3.7%), limonene (2.9%).

Fifty-four components of the KT200 sample were identified representing 97.4% of the oil. α-Terpineol (35%) and 1,8-cineole (11.6%) were the major compounds and sixteen volatiles are higher than 1%. Other major compounds are T-cadinol (9.4%), spathulenol (4.4%), camphene (3.4%), (*E*)-β-ocimene (3.1%), caryophyllene oxide (3.1%), β-caryophyllene (3.0%), γ-cadinene (2.7%), α-pinene (2.2%).

Forty-three compounds of the KT201 sample were detected representing 84.3% of the oil and α-pinene (18.4%) and β-caryophyllene (8.9%) were the major components. Spathulenol (6.3%), germacrene D (4.8%), β-bourbonene (4.5%), caryophyllene oxide (4.2%), limonene (3.9%), *trans*-verbenol (2.9%), *cis*-verbenol (2.7%) were the other major volatiles.

A previous study reported that the essential oil of aerial parts at the flowering stage of *T. sipyleus* subsp. *sipyleus* var. *rosulans* collected from İspir, Erzurum was characterized with carvacrol (30.0%), thymol (14.5%), *p*-cymene, α-terpinyl acetate and linalool as main components [21]. Akçin (2008) published that the volatile constituents of the oil of *T. sipyleus* subsp. *rosulans* samples collected from different regions showed significant differences. In the essential oil of Kastamonu sample, higher levels of myrcene (5.2%), 1,8-cineole (16.6%) were found while germacrene D-4-ol (8.2%), α-cadinol (6.4 %), germacrene D (5.21%), (*Z*)-β-farnesene (4.4%) and bicyclogermacrene (4.0%) in the samples from Çorum. In general, β-caryophyllene (6.8–14.2%), linalool (0.1–22.5%), 1,8-cineole (0.1–16.6%), α-terpineol (2.2–7.0%), caryophyllene oxide (1.9–8.1%), germacrene D (1.4–5.2%) and spathulenol (2.1–4.8%) were detected as major compounds in the samples [22]. Tepe et al. (2005) reported that 47 constituents were identified representing 98.7% of the oil of *Thymus sipyleus* subsp. *sipyleus* var. *rosulans* at flowering stage collected from Kangal, Sivas. This oil is characterised by the high monoterpene fraction (94.0%) and carvacrol (58.1%), thymol (20.5%) and *p*-cymene (4.1%) and γ-terpinene (4.4%) as main constituents [23].

Sixty-four total components of three *T. sipyleus* subsp. *sipyleus* var. *davisianus* samples were identified. Forty-six components of the KT196 sample were detected representing 90.5% of the oil, geranial (30.3%) and neral (19.6%) were the major compounds. Fourteen volatiles are higher than 1% and other notable components are caryophyllene oxide (6.2%), β-caryophyllene (5.1%), borneol (2.8%), 1-octen-3-ol (2.8%).

Thirty-one components of the KT197 sample were identified representing 96.0% of the oil. 1,8-cineole (31.1%) and β-caryophyllene (14.6%) were the major components. Sixteen volatiles are higher than 1% and other major compounds are *p*-cymene (12.4%), β-pinene (4.4%), linalool (6.4%), terpinen-4-ol (3.3%), sabinene (2.8%), α-thujene (2.6%) and α-pinene (2.1%).

Forty five components of the KT198 sample were detected representing 96.2% of the oil. The major compounds are α-terpineol (19.8%) and geranial (11.1%). Other major volatiles are β-caryophyllene (8.2%), 1,8-cineole (7.4%), neral (6.6%), myrcene (4.9%), linalool (4.7%), borneol (4.0%) and caryophyllene oxide (3.5%). Contents of nineteen compounds are higher than 1%.

In a previous study, Meriçli and Tanker (1986) reported that the essential oil of *T. sipyleus* subsp. *sipyleus* var. *davisianus* collected from Tefenni was rich in geranial (32.1%) [24]. The essential oil of aerial parts at full flowering stage of *T. sipyleus* subsp. *sipyleus* var. *davisianus* collected from Uşak was characterized with thymol (38.3%) and carvacrol (37.9%) among identified fourteen constituents [25].

Totally eighty volatile compounds of three *T. sipyleus* subsp. *sipyleus* var. *sipyleus* samples were identified by GC and GC-MS systems. Sixty-one volatile compounds of the KT190 sample were identified representing 98.5% of the oil. The major compounds are *p*-cymene (21.8%) and 1,8-cineole (11.2%). Eighteen volatiles are higher than 1% and other major compounds are carvacrol (9.1%), γ-terpinene (7.5%), borneol (7.6%), β-caryophyllene (7.1%) and camphene (4.5%).

Fifty-six volatiles of the KT191 sample were detected representing 99.0% of the oil, α-terpineol (35.8%) and carvacrol (20.5%) were the main compounds. Thirteen volatiles are higher than 1% and other major compounds are *p*-cymene (8.7%), γ-terpinene (4.2%), camphene (3.6%), myrcene (3.4%).

Fifty-four volatiles of the KT192 sample were identified representing 97.8% of the oil and the major compounds are carvacrol (18.2%) and 1,8-cineole (11.6%). The contents of fifteen volatiles are higher than 1% and other major compounds are *p*-cymene (9.2%), camphor (8.3%), camphene (7.2%), 3-octanol (5.8%), β-caryophyllene (5.0%), borneol (4.9%).

Demirci et al. (2018) reported that the essential oil of air dried and crushed aerial parts of *T. sipyleus* subsp. *sipyleus* var. *sipyleus* collected from Ulaş, Sivas, was characterized by high amount of thymol (66.2%), followed by *p*-cymene (9.4%), and γ-terpinene (9.2%) [11]. In another study, the chemical composition of T. *sipyleus* subsp. *sipyleus* var. *sipyleus* essential oil which originated from different regions (Denizli, Afyon, Ankara, Muğla, Konya) contained geranial (8.4%–37.0%), neral (3.1%–25.6%), linalool (21.8%), and α-terpineol+isoborneol (25.5%) as main components [26]. In a study published by Tepe et al. (2005), the aerial parts of *T. sipyleus* subsp. *sipyleus* var. *sipyleus* collected from Düziçi, Osmaniye were subjected to water distillation. Seventy-one volatile compounds were identified representing 92.5% of the total oil. The major compounds were borneol (11.2%), α-muurolol (9.2%), β-caryophyllene (7.6%), geranial (7.3%) and neral (5.4%) [23]. Pekgözlü and Özcan (2018) found citronellol as major compound in the SDE sample of *T. sipyleus* var. *sipyleus* leaves collected from Büğdüz, Burdur [27].

To sum up, published studies and our present study have generally shown a great deal of variability and diversity. *Thymus sipyleus* subsp. *sipyleus* var. *sipyleus* samples collected from three regions of Elmali were characterized with different major compounds such as 1,8-cineole, *p*-cymene, α-terpineol and carvacrol. The major volatile compounds in *T. sipyleus* subsp. *sipyleus* var. *davisianus* samples (three different sites of Saklıkent) were identified as 1,8-cineole, *p*-cymene, β-caryophyllene, geranial, and α-terpineol with different percentage amounts. *T. sipyleus* subsp. *rosulans* (three different sites of Gazipaşa) samples with major constituents as α-pinene, 1,8-cineole, β-caryophyllene, α-terpineol were identified.

## 4. Conclusion

Thymol is the major compound of most *Thymus* species. According to published data and our present study, chemical polymorphism has been found among the *Thymus sipyleus* subspecies even though the samples were collected from the same region. *Thymus* populations collected from Turkey have a greater variation of the major components in volatile oils. The variation of volatile oil composition has great importance due to its uses as food and in food processes. The results obtained here suggest that the growing conditions of thyme may alter the volatile oil content and composition.

## Figures and Tables

**Table 1:** Data on GPS and locations of the plant materials

Taxon	Location	Coordinate	Altitude
*T. sipyleus* subsp. *sipyleus* var. *sipyleus*	Elmalı	N36 43.581 E29 43.531	1599 m
*T. sipyleus* subsp. *sipyleus* var. *davisianus*	Saklıkent	N36 49.921 E30 19.600	2023 m
*T. sipyleus* subsp*. sipyleus* var. *rosulans*	Gazipaşa	N36 25.167 E32 33.113	2005 m

**Table 2:** Volatile compounds of *Thymus sipyleus* subspecies.

RR^I^a	RRI^b^	Compound	*T. sipyleus* subsp.*rosulans* %			*T. sipyleus* subsp. *sipyleus* var. *davisianus* %			*T. sipyleus* subsp*. sipyleus* var*. sipyleus* %			IM
			A	A	A	B	B	B	C	C	C	
			199	200	201	196	197	198	190	191	192	
1000	1000 ^c^	Decane	0.2	-	-	0.1	**-**	**-**	**-**	**-**	**-**	t_R_, MS
1014	998–1029 ^d^	Tricyclene	-	0.1	-	tr	-	0.1	0.1	0.1	0.2	MS
1032	1008–1039 ^d^	**α-Pinene**	1.4	2.2	**18.4**	0.9	2.1	1.4	1.5	1.4	2.0	t_R_, MS
1035	1012–1039 ^d^	α-Thujene	-	-	-	-	2.6	-	1.5	1.0	1.0	MS
1076	1043–1086 ^d^	Camphene	0.9	3.4	1.7	1.7	-	3.4	4.5	3.6	7.2	t_R_, MS
1118	1085–1130 ^d^	β-Pinene	0.8	1.0	0.9	0.2	4.4	0.9	1.0	0.3	0.8	t_R_, MS
1132	1098–1140 ^d^	Sabinene	0.4	0.9	-	-	2.8	0.5	0.3	0.2	0.3	t_R_, MS
1136	1109–1137 ^d^	Thuja-2,4(10)-diene	-	-	1.5	-	-	-	**-**	**-**	**-**	MS
1174	1140–1175 ^d^	Myrcene	0.8	0.7	1.9	0.9	1.0	4.9	1.5	3.4	1.0	t_R_, MS
1188	1154–1195 ^d^	α-Terpinene	0.3	-	-	-	0.9	-	1.6	0.8	0.3	t_R_, MS
1195	1167–1197 ^d^	Dehydro 1,8-cineole	-	0.1	-	**-**	**-**	**-**	-	0.1	-	t_R_, MS
1203	1178–1219 ^d^	Limonene	2.9	1.8	3.9	tr	1.5	1.7	0.7	0.8	0.7	t_R_, MS
1213	1186–1231 ^d^	**1,8-Cineole**	8.7	**11.6**	2.0	0.9	**31.1**	7.4	**11.2**	-	**11.6**	t_R_, MS
1215	1215 ^e^	*p*-Mentha-1,3,6-triene	-	-	0.9	0.4	-	-	**-**	**-**	**-**	MS
1218	1188–1233 ^d^	β-Phellandrene	-	-	-	-	-	-	**-**	0.2	**-**	t_R_, MS
1244	1213–1249 ^d^	2-Pentyl furan	-	-	-	tr	-	-	**-**	**-**	**-**	MS
1246	1211–1251 ^d^	(*Z*)-β-Ocimene	-	0.3	0.5	**-**	**-**	**-**	0.1	-	-	t_R_, MS
1255	1222–1266 ^d^	γ-Terpinene	0.7	0.2	tr	-	1.9	0.2	7.5	4.2	2.4	t_R_, MS
1266	1232–1267 ^d^	(*E*)-β-Ocimene	1.2	3.1	1.8	-	1.8	0.6	2.2	-	-	t_R_, MS
1267	1230–1280 ^d^	3-Octanone	1.0	1.8	1.0	0.8	-	1.5	-	0.4	1.5	t_R_, MS
1280	1246–1291 ^d^	***p*** **-Cymene**	1.1	0.4	2.4	0.7	**12.4**	0.5	**21.8**	8.7	9.2	t_R_, MS
1290	1261–1300 ^d^	Terpinolene	-	0.2	-	-	tr	0.2	0.3	-	tr	t_R_, MS
1296	1267–1312 ^d^	Octanal	-	-	-	tr	-	-	**-**	**-**	**-**	t_R_, MS
1348	1317–1357 ^d^	6-methyl-5-hepten-2-one	-	-	-	0.9	-	0.3	**-**	**-**	**-**	MS
1382	1334–1394 ^d^	*cis*-Alloocimene	-	-	-	-	-	-	0.1	**-**	**-**	MS
1393	1372–1408 ^d^	3-Octanol	0.4	0.7	-	0.4	0.5	1.1	0.1	0.8	5.8	MS
1400	1370–1414 ^d^	Nonanal	0.9	0.1	-	0.3	1.1	0.8	tr	tr	-	MS
1413	1413 ^e^	Rosefuran	-	-	-	1.1	-		**-**	**-**	**-**	MS
1429	1405–1431 ^d^	Perillene	-	-	-	0.4	-	0.2	**-**	**-**	**-**	MS
1449	1412–1457 ^d^	*p*-Cymenene	-	-	-	tr	-	**-**	tr	-	-	MS
1452	1411–1465 ^d^	1-Octen-3-ol	1.0	1.2	1.7	2.8	0.8	1.3	1.9	0.5	0.7	t_R_, MS
1460	1460 ^f^	2,6-Dimethyl-1,3(*E*), 5(*E*)-7-octatetraene	-	0.2	-	**-**	**-**	**-**	tr	-	-	MS
1461	1463 ^n^	(*E*)-2-hexenyl butyrate	-	**-**	**-**	**-**	-	**-**	tr	**-**	tr	MS
1466	1438–1480 ^d^	α-Cubebene	-	**-**	**-**	**-**	-	**-**	-	**-**	0.4	MS
1474	1425–1478 ^d^ 1474 ^f^	*trans*-Sabinene hydrate	0.9	tr	-	-	1.1	-	1.5	0.5		MS
1478	1478 ^f^ 1479 ^h^	*cis-*Linalool oxide (fur.)	-	-	-	-	-	-	tr	-	-	MS
1493	1459–1500 ^d^	*α-*Ylangene	-	-	-	-	-	-	tr	-	-	MS
1495	1452–1513 ^d^	2-Ethyl hexanol	0.3	tr	-	-	0.4	tr	**-**	**-**	**-**	MS
1496	1471–1495 ^d^	Bicycloelemene	0.5	0.2	0.5	-	0.9	-	0.4	0.8	1.3	MS
1496	1495 ^h^	*cis, cis*-Photocitral	-	-	-	0.7	-	-	**-**	**-**	**-**	MS
1497	1462–1522 ^d^	α-Copaene	-	tr	0.3	-	-	-	0.1	-	-	MS
1519	1519 ^h^	*trans, trans*-Photocitral	-	-	-	1.4	-	0.7	**-**	**-**	**-**	MS
1532	1481–1537 ^d^	Camphor	1.5	0.1	2.6	0.2	-	-	0.1	tr	8.3	t_R_, MS
1535	1496–1546 ^d^	β-Bourbonene	1.4	0.3	4.5	0.6	0.3	0.3	0.1	0.1	0.3	t_R_, MS
1549	1518–1560 ^d^	β-Cubebene	-	-	0.3	-	-	-	**-**	**-**	**-**	MS
1553	1507–1564 ^d^	Linalool	0.7	0.2	0.3	0.7	6.4	4.7	0.3	0.5	0.4	t_R_, MS
1555	1557 ^g^	1-Nonen-3-ol	-	-	-	-	-	-	0.2	0.1	0.1	MS
1556	1526–1565 ^d^ 1556 ^f^	*cis*-Sabinene hydrate	-	-	-	-	-	-	0.3	0.2	0.2	MS
1562	1519–1574 ^d^	Octanol	-	-	-	-	0.2	-	tr	-	-	t_R_, MS
1571	1557–1625 ^d^	*trans*-*p*-Menth-2-en-1-ol	-	-	-	-	-	-	0.1	-	-	MS
1588	1588–1610 ^d^	Bornyl formate	-	0.1	-	-	-	-	**-**	**-**	**-**	MS
1589	1547–1589 ^d^	β-Ylangene	0.6	-	-	**-**	**-**	**-**	**-**	**-**	**-**	MS
1590	1549–1597 ^d^	Bornyl acetate	-	1.2	-	0.9	-	2.0	1.6	2.0	2.5	t_R_, MS
1600	1565–1608 ^d^	β-Elemene	1.0	tr	0.7	**-**	**-**	**-**	**-**	**-**	**-**	MS
1611	1564–1630 ^d^	Terpinen-4-ol	1.3	0.3	-	0.5	3.3	0.3	2.5	0.8	0.9	t_R_, MS
1612	1569–1632 ^d^	**β-Caryophyllene**	**14.2**	3.0	8.9	5.1	**14.6**	8.2	7.1	2.3	5.0	t_R_, MS
1624	1600–1650 ^d^	*trans*-Dihydrocarvone	-	-	-	-	-	-	-	0.4	0.2	t_R_, MS
1628	1583–1668 ^d^	Aromadendrene	-	-	-	-	-	-	0.4	0.2	0.5	MS
1645	1645 ^h^	*cis*-Dihydrocarvone	-	-	-	-	-	-	-	0.1	-	t_R_, MS
1663	1647–1668 ^d^	*cis*-Verbenol	-	-	2.7	**-**	**-**	**-**	**-**	**-**	**-**	MS
1661	1624–1668 ^d^	Alloaromadendrene	-	0.1	-	**-**	**-**	**-**	0.1	-	0.1	MS
1668	1627–1668 ^d^	(*Z*)-β-Farnesene	-	0.5	-	0.7	-	-	-	0.1	0.4	MS
1683	1665–1691 ^d^	*trans*-Verbenol	-	-	2.8	**-**	**-**	**-**	**-**	**-**	**-**	MS
1687	1637–1689 ^d^	α-Humulene	3.7	0.6	0.9	0.3	tr	0.4	0.7	0.2	tr	t_R_, MS
1694	1641–1706 ^d^	**Neral**	0.5	-	-	**19.6**	-	6.6	-	0.1	-	MS
1704	1655–1714 ^d^	γ-Muurolene	-	-	0.5	**-**	**-**	**-**	**-**	**-**	**-**	MS
1706	1659–1724 ^d^	**α-Terpineol**	0.7	**35.0**	-	0.3	1.3	**19.8**	3.2	**35.8**	0.8	t_R_, MS
1708	1708 ^c, h^ 1707 ^f^	Ledene	-	-	-	-	-	-	0.2	0.1	0.3	MS
1719	1653–1728 ^d^	Borneol	-	0.5	-	2.8	0.5	4.0	7.6	2.8	4.9	t_R_, MS
1725	1696–1735 ^d^	Verbenone	-	-	0.7	**-**	**-**	**-**	**-**	**-**	**-**	t_R_, MS
1726	1676–1726 ^d^	Germacrene D	0.8	0.4	4.8	-	0.3	0.3	0.3	0.3	0.5	MS
1730	1730 ^g^	δ-Guaiene	0.7	-	-	**-**	**-**	**-**	**-**	**-**	**-**	MS
1732	1732^m^	Bicyclosesquiphellandrene	-	-	0.5	**-**	**-**	**-**	-	-	tr	MS
1740	1698–1748 ^d^	β-Bisabolene	-	-	-	**-**	**-**	**-**	-	0.8	2.1	t_R_, MS
1740	1686–1753 ^d^	α-Muurolene	-	-	-	**-**	**-**	**-**	0.1	-	-	MS
1741	1680–1750 ^d^	**Geranial**	0.8	-	-	**30.3**	-	**11.1**	-	0.4	-	MS
1744	1696–1748 ^d^	α-Selinene	-	-	-	-	-	-	0.1	-	-	MS
1746	1744 ^m^	Selina-4(15),7(11)-diene	0.8	-	-	**-**	**-**	**-**	**-**	**-**	**-**	MS
1755	1692–1757 ^d^	Bicyclogermacrene	0.6	-	0.3	-	0.1	0.4	0.5	1.0	1.5	MS
1772	1734–1789 ^d^	Citronellol	-	-	-	1.4	-	0.5	**-**	**-**	**-**	t_R_, MS
1773	1722–1774 ^d^	δ-Cadinene	-	-	0.5	**-**	**-**	**-**	0.3	0.1	0.4	t_R_, MS
1776	1735–1782 ^d^	γ-Cadinene	-	2.7	0.2	**-**	**-**	**-**	0.5	0.1	0.3	MS
1797	1787 ^m^	Aromadendra-1(10), 4(15)-diene	-	-	-	**-**	**-**	**-**	-	-	tr	MS
1808	1752–1832 ^d^	Nerol	-	-	-	-	-	tr	**-**	**-**	**-**	t_R_, MS
1816	1734–1803 ^d^	α-Cadinene	-	tr	-	**-**	**-**	**-**	**-**	**-**	**-**	MS
1853	1800–1853 ^d^	*cis*-Calamenene	-	0.4	-	**-**	**-**	**-**	-	tr	tr	MS
1857	1795–1865 ^d^	Geraniol	-	-	-	tr	**-**	**-**	**-**	**-**	**-**	t_R_, MS
1864	1813–1865 ^d^	*p*-Cymen-8-ol	-	-	-	**-**	**-**	**-**	-	tr	-	MS
1868	1868 ^c, g, h^	(*E*)-Geranyl acetone	-	-	-	tr	**-**	**-**	**-**	**-**	**-**	t_R_, MS
1900	1900 ^p^	Nonadecane	-	-	-	**-**	**-**	**-**	tr	-	-	t_R_, MS
2008	1936–2023 ^d^	Caryophyllene oxide	6.2	3.1	4.2	6.2	-	3.5	0.7	0.5	0.6	t_R_, MS
2029	1963–2029 ^d^	Perilla alcohol	-	-	-	1.2	-	-	**-**	**-**	**-**	MS
2037	2016–2043 ^d^	Salvial-4(14)-en-1-one	0.3	tr	0.8	**-**	**-**	**-**	**-**	**-**	**-**	MS
2069	2000–2070 ^d^	Germacrene D-4-β-ol	-	-	-	**-**	**-**	**-**	-	-	tr	MS
2071	2003–2071 ^d^	Humulene epoxide II	1.4	0.5	-	**-**	**-**	**-**	0.1	tr	-	MS
2080	2019–2090 ^d^	Cubenol	-	1.1	-	**-**	**-**	**-**	0.1	tr	tr	MS
2096	2043–2103 ^d^	Elemol	-	-	-	**-**	**-**	**-**	tr	-	-	MS
2098	2049–2104 ^d^	Globulol	-	-	-	**-**	**-**	0.1	-	-	tr	MS
2123	2123 ^e^ 2130 ^k^	Salviadienol	-	0.2	1.1	**-**	**-**	**-**	**-**	**-**	**-**	MS
2144	2074–2150 ^d^	Spathulenol	7.0	4.4	6.3	0.3	0.7	2.7	0.8	1.2	2.0	t_R_, MS
2170	2090–2189 ^d^	b-Bisabolol	-	0.2	-	**-**	**-**	**-**	**-**	**-**	**-**	MS
2187	2136–2200 ^d^	T-Cadinol	-	9.4	-	0.3	-	-	0.4	0.1	0.3	MS
2198	2100–2205 ^d^	Thymol	tr	-	-	-	0.1	-	0.8	0.9	0.4	t_R_, MS
2219	2211 ^k^	Clovenol	-	-	-	-	-	tr	**-**	**-**	**-**	t_R_, MS
2239	2140–2246 ^d^	**Carvacrol**	0.7	tr	0.2	-	0.9	tr	9.1	**20.5**	**18.2**	t_R_, MS
2243	2243 ^e^	Torilenol	0.5	0.3	1.1	**-**	**-**	**-**	**-**	**-**	**-**	MS
2247	2247 ^f, g^	*trans*-α-Bergamotol	0.6	0.2	0.4	-	-	0.2	-	tr	0.1	MS
2255	2180–2255 ^d^	α-Cadinol	0.3	0.3	0.3	0.4	-	-	-	tr	0.1	t_R_, MS
2264	2218–2264 ^d^	**Intermedeol**	**13.2**	-	-	**-**	**-**	**-**	**-**	**-**	**-**	MS
2316	2316 ^g^	Caryophylladienol I	0.7	0.5	0.8	tr	-	0.2	tr	-	-	MS
2324	2324 ^c, f, g^	Caryophylladienol II	1.4	-	-	1.6	-	1.3	0.1	0.1	-	MS
2369	2351–2402 ^d^	Eudesma-4(15),7-diene-1-β-ol	1.1	0.3	1.7	**-**	**-**	**-**	**-**	**-**	**-**	MS
2389	2389 ^g, h^	Caryophyllenol I	1.3	0.6	0.6	1.5	-	1.0	0.1	0.1	-	MS
2392	2392 ^c, d, f, g^–2396^d^	Caryophyllenol II	1.6	0.7	1.0	1.0	-	0.9	0.1	0.1	-	MS
		**Total**	**90.0**	**97.4**	**84.3**	**90.5**	**96.0**	**96.2**	**98.5**	**99.0**	**97.8**	

RRI^a^: relative retention indices calculated against *n*-alkanes (C_8_ to C_25_). %: calculated from the FID chromatograms. RRI^b^: RRI from literature (c [28], d [29], e [30], f [31], g [32], h [33], k [34], m [35], n [36], p [37]) for polar column values. tr: trace (<0.1 %). IM: identification method. t_R_: identification based on the retention times (t_R_) of genuine compounds on the HP Innowax column. MS: identified on the basis of computer matching of the mass spectra with those of the in-house Baser Library of Essential Oil Constituents, Adams, MassFinder and Wiley libraries and comparison with literature data.
